# Resilience in Mild Cognitive Impairment (MCI): Examining the Level and the Associations of Resilience with Subjective Wellbeing and Negative Affect in Early and Late-Stage MCI

**DOI:** 10.3390/bs13100792

**Published:** 2023-09-23

**Authors:** Styliani Olympia Tsormpatzoudi, Despina Moraitou, Vasileios Papaliagkas, Christos Pezirkianidis, Magda Tsolaki

**Affiliations:** 1 Neurosciences and Neurodegenerative Diseases, Postgraduate Course, Medical School, Faculty of Health Sciences, Aristotle University, 54124 Thessaloniki, Greece; tsolakim1@gmail.com; 2 Laboratory of Psychology, Department of Experimental and Cognitive Psychology, School of Psychology, Faculty of Philosophy, Aristotle University, 54124 Thessaloniki, Greece; demorait@psy.auth.gr; 3 Laboratory of Neurodegenerative Diseases, Center for Interdisciplinary Research and Innovation (CIRI-AUTH), Balkan Center, Aristotle University, 10th km Thessaloniki-Thermi, 54124 Thessaloniki, Greece; 4 Department of Biomedical Sciences, School of Health Sciences, International Hellenic University, 57400 Thessaloniki, Greece; vpapaliagkas@gmail.com; 5 Laboratory of Positive Psychology, Panteion University of Social & Political Sciences, Syggrou Ave. 136, 17671 Athens, Greece; pezir@panteion.gr; 6 Greek Association of Alzheimer’s Disease and Related Disorders (GAADRD), 54643 Thessaloniki, Greece

**Keywords:** resilience, positive emotions, engagement, accomplishment, negative emotions, depression, anxiety, stress, early MCI, late MCI

## Abstract

The current study examines the relationship between the cognitive state of participants [healthy-early mild cognitive impairment (MCI)–late MCI], some subjective wellbeing factors (positive emotions, engagement, positive relationships, meaning in life, accomplishment, and negative emotions), and negative psychological outcomes (depression, anxiety, stress), as well as psychological resilience. We expected that people with advanced MCI would perceive increased negative psychological outcomes, poorer psychological resilience, and lower levels of subjective wellbeing in contrast to early MCI and healthy participants. The study involved 30 healthy, 31 early, and 28 late MCI individuals. A series of questionnaires have been applied to assess the aforementioned constructs. To examine the hypotheses of the study, path analysis (EQS program) was applied. Results showed that early MCI persons maintain the same levels of positive emotions and feelings of accomplishment with healthy peers. Late-stage patients present those feelings in a diminished form, which adversely impacts psychological resilience. Individuals with early and late MCI exhibit negative emotions and stress that impact their resilience; however, those with early MCI experience greater stress, negative emotions, depression, and anxiety. These findings may be utilized to design psychological interventions for resilience enhancement and support brain health in elderly adults who are at risk of neurodegeneration.

## 1. Introduction

### 1.1. Mild Cognitive Impairment 

Mild cognitive impairment (MCI) is characterized as a syndrome in which the individual exhibits a discreet cognitive decline on neuropsychological tests compared to people of the same age and educational level, but this impairment does not have a notable impact on everyday functioning [1,2]. According to DSM-5 [3], specific subtypes of mild cognitive impairment (MCI) are not outlined, as the focus is primarily on the overall classification and diagnosis of neurocognitive disorders, encompassing MCI. However, beyond the DSM-5, alternative research has proposed a total of four subtypes of MCI, considering whether the symptoms are amnestic or non-amnestic, and whether they affect a single domain or multiple domains [4].

According to the seminal work by Petersen and his colleagues [5], the diagnostic criteria for MCI encompass a range of pivotal elements. Firstly, it is imperative for individuals to exhibit demonstrable evidence of cognitive decline. Secondly, the presence of objective cognitive impairment should be discernible through the utilization of standardized cognitive tests, indicating deficits surpassing those anticipated for an individual’s corresponding age and educational background (>1.5 SD) in one area of cognitive function. Thirdly, the functional capacities and execution of daily living activities ought to remain relatively preserved, allowing individuals to autonomously carry out their customary tasks. Lastly, it is crucial to note that the established criteria mandate the exclusion of dementia as a diagnosis, as MCI denotes a crucial transitional stage positioned between the realms of normative cognitive aging and dementia. The criteria delineated by Petersen and colleagues in 1999 [5] constitute a valuable and indispensable framework for effectively identifying and accurately diagnosing individuals presenting with MCI.

The rising rates of MCI prevalence around the world present dire warnings to scientists and health professionals about early detection and intervention in aged people. Studies have gradually shed light on the potential diagnosis and the causal mechanisms and try to identify paths of progression on the cognitive decline to map the broad range of clinical displays. 

### 1.2. Psychological Resilience

The American Psychological Association [6] defines resilience as “the process of adapting well in the face of adversity, trauma, tragedy, threats or even significant sources of stress” (paragraph 1, pp. 1). Two important concepts included in almost every definition of psychological resilience are “adversities” and “positive adaptation” [7]. 

A dominant theoretical framework of psychological resilience comes from the field of developmental psychology, in which Garmezy and his colleagues [8] are credited with pioneering studies. Their research focused on the potential psychopathological traits of children raised by psychologically disturbed parents. The findings showed that some of those children could overcome the challenges of their environments even if they were exposed to overwhelming adversities [9]. Further, psychiatrist Rutter [10] supported the notion that psychological resilience is not a broad personality trait. He emphasizes that resilience is a multidimensional concept that can differ from one person, situation, or period to another, and can be one of several outcomes that come from a single experience. Luthar and his colleagues [11], embracing the theoretical frameworks of Garmezy and Streitman [12] and Rutter [13], argue that the combination of protective mechanisms with the countervailing factors is the component that makes children adaptive other than the environmental challenges. 

In contemporary understanding, resilience is no longer perceived as an inherent personality trait but rather as a dynamic process, akin to the adaptability of human behavior [14,15]. Recent research findings suggest that as individuals advance in age, their resilience is contingent upon their initial capacity to effectively navigate environmental challenges [16,17], but it can also evolve across the lifespan due to normative developmental and aging processes [18,19]. Among the various challenges faced by older adults, a notable example is the diagnosis of a neurodegenerative disease. The level of resilience exhibited in response to such challenges is linked to a combination of factors, including protective elements such as positivity, optimism, and competence, coupled with risk factors, including negative affective states like depression, anxiety, and stress. The interplay of these factors collectively determines whether an individual’s resilience levels will be higher or lower.

### 1.3. Resilience and Subjective Wellbeing in Older Adults

The increasing population of older adults [20] is a pressing concern that necessitates focused research and attention toward their wellbeing. Subjective wellbeing, a multifaceted construct, encompasses several critical components such as positive emotions, engagement, relationships, life meaning, and accomplishment, all of which contribute significantly to an individual’s overall wellbeing [21]. Empirical evidence suggests that the ability to experience positive emotions effectively aids highly resilient individuals in recovering from daily stressors [22], underscoring the pivotal role of positive affect in the resilience of older adults [23,24]. However, when cognitive impairment occurs, positive as well as negative subjective wellbeing indicators seem to depend on the rate of impairment [25]. 

Another essential aspect in pursuing high levels of subjective wellbeing in aging is achievements, which include setting goals and having the capacity to successfully accomplish them [26]. Older people who continue to set goals and remain active to accomplish them—primarily by maintaining high levels of physical and emotional health and thinking positively about the future [27]—have greater subjective wellbeing benefits, particularly from the satisfaction of independence and competency [28]. Camitan and Bajin [29] found that accomplishment positively predicts resilience in healthy adults. Nevertheless, there is a scarcity of scientific research regarding the accomplishments in cognitively impaired older adults and the association with resilience, thus indicating the need for further in-depth exploration in this subject.

Finally, engagement is a component that contributes to a person’s competency to achieve their goals and, generally, to influence subjective wellbeing conditions. Csikszentmihalyi [30] defines engagement as a profound involvement in the work at hand that includes sentiments of enjoyment. According to McFadden and Basting [31], dementia patients who engage in creative activities increase their resilience by identifying and expressing their abilities. However, engagement has not been investigated adequately in MCI. 

### 1.4. Resilience and Negative Psychological Outcomes in Older Adults

In contrast to the previous factors that are positively associated with resilience, there are some psychological constructs that appear to be associated with the failure of resilience. A negative correlation is shown between resilience and depression [32,33], anxiety [34], and negative emotions [35], whereas negative emotions can negatively predict resiliency [29]. Indicatively, major depressive disorder is found in up to 10% of people over 60 [36] and those with MCI often experience depression, anxiety, and apathy [37]. The neurodegenerative foundation, along with a concurrent negative psychological state, may account for more severe and faster cognitive deficits, while these patients progress to dementia more often than MCI people without these compounding factors [37,38,39]. 

Furthermore, anxiety is frequently expressed in older adults as a psychological indication of the dread of losing control, dissatisfaction, and restlessness [40], while stress is more associated with physiological responses [41,42]. According to Rozzini and his colleagues [40], anxiety disturbances in MCI are independently connected to impaired people’s levels of executive functions, implying that anxious patients perform poorly on the tasks of daily life that require planning, organization, and initiation. In contrast, Jain and colleagues [43] revealed in longitudinal studies that perceived stress is a significant mediator in the relation between anxiety and MCI. It seems that, among healthy individuals, resilience had an indirect influence on stress response, and it can eventually modulate final cortisol levels through active coping [44].

In summary, it is evident that for individuals facing mild cognitive impairment (MCI), resilience assumes a pivotal role in addressing cognitive alterations and fostering a positive perspective. Resilience equips these individuals with the tools to navigate the intricate emotional and psychological challenges that accompany MCI, ultimately bolstering their capacity to confront difficulties while preserving their subjective wellbeing. However, it is important to acknowledge that current research findings concerning the interplay between psychological resilience, the multifaceted dimensions of subjective wellbeing, and negative psychological affect do not yet offer a fully comprehensive understanding of these topics [45].

### 1.5. Aim and Hypotheses of the Study

Previous research has examined subjective wellbeing factors and psychological resilience independently or in populations other than people with MCI. However, the previous variables have not been studied in a single research design and there are not enough data in the literature to explain the connection between the degree of a person’s neurodegeneration, their subjective wellbeing, the negative psychological outcomes, and resilience. Therefore, the aim of the present study was to examine resilience in MCI. Specifically, we were interested in whether people in early-stage MCI differ from others who are classified as part of the advanced MCI stage, as well as healthy adults, in terms of their levels of psychological resilience and its relationship with subjective wellbeing and negative affect (depression, anxiety, and stress). 

The first hypothesis of the study was that late-stage MCI people would exhibit lower levels of resilience and a profile of different associations between resilience, subjective wellbeing, and negative affect, when compared to early-stage MCI people and cognitively healthy individuals (H_1_). 

In the same vein, the second hypothesis of the study was that early-stage MCI people would exhibit lower levels of resilience and a profile of different associations between resilience, subjective wellbeing, and negative affect, when compared to healthy controls (H_2_).

## 2. Methods

### 2.1. Participants

Participants with MCI were recruited from the daily programs of people who have already been diagnosed with mild cognitive impairment at the Day Centers of Alzheimer Hellas “Saint Helen” and “Saint Ioannis” in Thessaloniki from September 2022 to May 2023. The tests that were used for the diagnosis of the MCI included the Montreal Cognitive Assessment Scale [46,47], the Functional Cognitive Assessment [48], the Mini Mental State Examination [49,50], the Global Deterioration Scale (GDS) [51], the Neuropsychiatric Inventory [52,53], the Geriatric Depression Scale [54,55], the Beck Depression Inventory [56], the Short Anxiety Screening Test [57,58], and the Beck Anxiety Inventory [59]. Standardized assessments for general cognitive and functional abilities, memory capacity, linguistic ability, executive functions, and attention were also employed. 

The eligibility requirements for MCI diagnosis were established based on the DSM-5 criteria for mild neurocognitive disorders. Diagnostic evaluations, including neurological examination, neuropsychological and neuropsychiatric assessments, neuroimaging, and blood tests, were conducted for every visitor to the Day Centers. The criteria for the MCI diagnosis included a diagnosis of minor neurocognitive disorders as it is stated in DSM-5 and a minimum total score of 24 on the Mini-Mental State Examination (MMSE). Moreover, the visitors of the centers are classified as stage 3 of the disease, based on the Global Deterioration Scale, and score at least 1.5 standard deviations (SDs) below the average for age and education in at least one cognitive domain, as determined by the utilized neuropsychological tests. Finally, specialized healthcare professionals from Alzheimer Hellas, who are recognized as experts in neurocognitive disorders, have reached a consensus for the diagnosis. 

For the purposes of the present study, MCI patients were divided into two groups in accordance with their scores of the screening test “Shapes” from the “Doors and People” battery [60,61]. The range of the scores for the late-stage MCI group was 0–21 and for the early-stage MCI group, 22–27, and patients were at least 60 years old. Individuals with scores higher than 27 or who were younger than 60 years old were excluded from the study. 

A total of 78 patients with MCI were assessed for eligibility. Of those, 19 potential participants did not meet the inclusion criteria or did not complete both parts of the study, due to illness or other reasons. Eventually, 59 patients took part in the study, 28 late-stage MCI and 31 early-stage MCI. From the 32 healthy controls (potential participants), 2 were excluded due to the criteria determined for them. Thus, the control group of the study consisted of the remaining 30 participants. 

The final sample consisted of 31 early MCI patients, 28 late MCI patients, and 30 cognitively healthy older participants. The control group comprised of healthy individuals of the same age. These individuals were informed about the study’s objectives and consented to participate. They were first assessed in a neuropsychological evaluation using MoCA [46,47,62] to ensure that they had no impairment in cognitive performance, and then they were given identical questionnaires to that of the other two groups. The inclusion criteria for healthy participants were: being older than 60 years of age and having a MoCA score higher than 26 (for less than 12 years of education) or 27 (for more than 12 years of education). Participants that did not meet the criteria were excluded. Table 1 shows in detail the descriptions of the sample. The three groups were matched according to gender (χ^2^ = 2.37; *p* = 0.305) and age (χ^2^ = 63.83; *p* = 0.126), but notable distinctions were observed in their educational background (χ^2^ = 25.71; *p* < 0.001), which was divided into two categories: low educational level (≤9 years of education) and middle to high educational level (>9 years of education). As shown, low educational level is mainly represented in the late MCI group, compared to the other two groups. 

### 2.2. Procedure

The study was executed in person and online. In the first scenario, the participants met with the researcher in a quiet room in the day centers of the Greek Association of Alzheimer’s Disease and Related Disorders, who briefed them about the study’s goals, their voluntary involvement and that they would receive no monetary compensation. Then, they were assessed for MCI severity with the screening test and handed a set of questions to complete. The participants were not forced to provide any answer which they were uncomfortable to share and were allowed to end their participation at any level of the study. 

Participants who completed the online questionnaire were approached by the organization’s virtual classes and were given the same information about the study. Following this, they were provided a link to the questionnaire (Google Forms), which potential applicants would complete. Participants provided their agreement to be contacted for the second part of the MCI severity screening, which was held via webcam at a set time and day by submitting some personal information (via e-mail). 

For both sampling methods, the duration of the questionnaire’s administration lasted about 20–25 min and the completion was generally conducted without any problem, except for a few cases where participants mentioned a slight discomfort and tiredness. 

### 2.3. Ethics

The study was approved by the Scientific and Ethics Committee of Alzheimer Hellas (81/14-09-2022), adhering to the guidelines of the new General Data Protection Regulation (EU) 2016/679 enacted by the European Parliament and the Council on 27 April 2016. This regulation ensures the protection of individuals’ personal data during processing and promotes the unrestricted movement of such data. Furthermore, the study abided the principles outlined in the Helsinki Declaration. 

During the initial clinical appointment, all study participants read the information sheet and signed the informed consent, stating that the research team has permission to use their anonymized personal data, such as gender, age, and education, as well as their performance in the neuropsychological tests, for research purposes. By participating in the study, individuals were also informed that they would be asked to provide certain personal information (e-mail addresses) to be contacted for the second part of the study, which would only be used for this reason and were discarded after the study’s completion. Participants were permitted to withdraw at any time during the study without penalty. 

### 2.4. Measures

#### 2.4.1. Screening Tests

##### MoCA

The Montreal Cognitive Assessment (MoCA) is one of the most well-adopted screening tools worldwide in detecting neurodegenerative diseases. It takes about 10–15 min to be administered and is sensitive to MCI detection in people older than 60 years [46]. The MoCA scores are not affected by gender or age [63], but are affected by educational level [64,65]. The cut-off scores for healthy adults in Greece are 26 for 7–12 and 27 for >13 years of education with good sensitivity (77.6%) and specificity (74.7%), whereas for less than 6 years of education, the cut-off is 23, resulting also in good sensitivity (71.4%) and specificity (84.2%) [47]. In the present study, the Greek version of MoCA (http://www.mocatest.org/wp-content/uploads/2015/testsinstructions/MoCA-Test-Greek.pdf (accessed on 20 September 2022)) has been used for screening the cognitive functioning of healthy people. MoCA assesses short-term memory (5 min delayed recall of 5 words), visuospatial abilities (clock-drawing test, 3D cube copy), attention abilities (detection using tapping, a serial subtraction task, and digits forward/backward), working memory, aspects of executive functioning (short trail making test, phonemic fluency task, and verbal abstraction tasks), language deficit (naming, repetition task), and orientation (time and space nomination). 

##### Doors and People: Subtest Shapes

“Door and People” is a four-part test that examines the episodic memory of diverse groups of individuals, including MCI and dementia patients [60,66,67]. The ΜCI patients only completed the Shapes subtest as a means of screening for the severity of their cognitive impairment. The Shapes test consists of four drawings and assesses both immediate and delayed recall. The participant has a maximum of three trials to accurately recreate the drawings, with each trial worth three points (score range: 0–36). For the Greek population aged 65–80 years old, the score for healthy adults was above 27, for early MCI 21–27, and for late MCI less than 21 [61].

#### 2.4.2. Main Research Tools

##### Brief Resilience Scale 

The Brief Resilience Scale (BRS) is a 6-item scale measuring resilience, developed by Smith, Dalen, Wiggins, Tooley, Christopher, and Bernard [68], that focuses on the capacity to recover from stress and adversity. It has a two-factor structure with Cronbach’s alpha values ranging from 0.80 to 0.91 across five samples [34,69,70,71,72]. The scale was translated using the translation/back-translation process and adapted in Greek [73,74]. The responses are given on a Likert-type scale, with scores from 1 to 5, with 1 being “strongly disagree”. To prevent the desirability response bias, Smith et al. [68] suggest the reversion of the items 2, 4, and 6 in statistical analysis.

##### The PERMA Profiler

Butler and Kern [26] constructed the PERMA Profiler based on Seligman’s [21] model of subjective wellbeing. It consists of 23 items and examines the five pillars of subjective wellbeing according to the theory (self-reports about positive emotions, engagement, positive relationships, meaning in life, accomplishment) as well as an overall subjective wellbeing score. It also assesses three additional factors: negative emotions, loneliness, and physical health. Pezirkianidis et al. [75] translated and adapted the questionnaire into Greek. The results also revealed adequate internal consistency and test–retest reliability for the overall subjective wellbeing items and almost all subjective wellbeing components. The Greek version of the PERMA Profiler exhibited high convergent validity with a variety of subjective wellbeing indices as well as discriminant validity with psychological symptoms and adverse emotional experiences. The responses are based on an 11-point Likert-type scale ranging from 0 (“never”, “not at all”, “terrible”) to 10 (“always”, “completely”, “excellent”).

##### DASS-21

The Depression Anxiety Stress Scale 21 (DASS-21) is the short version of DASS-42 [76] and represents an underlying three-factor structure [77]. The scale is a self-report questionnaire and measures anxiety, depression, and stress in clinical [78] and non-clinical populations [79]. Lyrakos et al. [80] examined the psychometric properties of the scale in Greek. The questions are scored on a Likert-type scale with 4 possible answers (0 = “not true for me” to 3 = “totally true for me”). Overall, DASS-21 has strong convergent and discriminant validity, as well as good internal consistency for each of the three factors, with Cronbach’s alpha values of 0.85, 0.84, and 0.84 for depression, anxiety, and stress, respectively, and Spearman–Brown values of 0.84, 0.83, and 0.85 [81]. 

##### Demographic Variables

Regarding demographics, age, gender, and education level were recorded. This information was filled out individually by the participants. 

### 2.5. Statistics

The statistical analysis was performed with the use of IBM SPSS Statistics for Windows version 28 (IBM; SPSS Statistics for Windows, Version 28.0. Armonk, NY, USA: IBM Corp 28.0) and the statistical significance was set at *p* < 0.05 level. To analyze the data, descriptive and inferential statistics were performed. The analyses carried out were (a) ANOVA, (b) Pearson Correlations/ Spearman’s rho, and (c) path analysis (EQS version 6.4 statistical software). 

At first, ANOVA was used to examine if there are differences in the resilience levels of the three different groups. Levene’s test assessed the equality of variances and partial eta-squared (η^2^) was used to measure the magnitude of the effects observed. To conduct additional comparisons between groups after the initial analysis, the researchers employed Bonferroni correction. 

Pearson Correlations were employed between the variables of the study to test the statistically significant relationships. Moreover, some variables did not follow the normal distribution and for this reason, Spearman’s rho correlations were conducted.

To examine the differences in the ‘profile’ of the relationships between resilience and the other variables in the three groups of the study, path analysis was conducted for each group. For the path analysis, the EQS program v.6.4 was used [82] to examine the directional relationships between subjective wellbeing (PERMA dimensions: positive emotions, engagement, positive relationships, meaning in life, accomplishment) and resilience. Another path was conducted between negative affect (depression, anxiety, stress) as well as negative emotions (from the PERMA Profiler) and resilience. ‘Diagnostic group’ was used as a predictive variable with two values representing two groups, respectively, in each path model confirmed. 

In assessing the fit between a model and the data, several indicators are considered. One such indicator is the χ^2^, where a non-significance level (*p* < 0.05) suggests a good fit to the data [82].

Another indicator, the Comparative Fit Index (CFI), compares the observed data with a hypothesized measurement model. A CFI value greater than 0.90 indicates an adequate fit of the model to the data, while values approaching 1.00 suggest a very good fit [83].

The Standardized Root Mean Square Residual (SRMR) index is an additional measure used to assess how well a model fits the observed data. The SRMR evaluates the difference between the actual data and the predicted values from the model, considering the standardization of residuals. A smaller SRMR value indicates a better fit of the model to the data, with values closer to zero indicating a more accurate fit.

An additional indicator is the Root-Mean-Squared Error of Approximation (RMSEA). A value below 0.05 indicates a good fit of the model to the data. However, if the RMSEA falls between 0.06 and 0.08, it suggests a reasonable approximation error that is still acceptable. 

It is important to note that the reliability of the RMSEA value is influenced by the sample size. In cases where the sample size is small (less than 100), the RMSEA value tends to be less precise, as reflected by the wider confidence interval range (90% CI). Therefore, caution is advised when interpreting the RMSEA as a model fit index, particularly for small sample sizes (n < 100) [84].

Besides the path analysis for the normally distributed data, robust path analysis was conducted between the negative affect and resilience variables. In this case, the χ^2^ index was replaced with the Satorra–Bentler (S–B) χ^2^ index, whereas SRMR cannot be calculated with this method.

By considering these statistical measures, we can assess the goodness of fit between our model and the data in a rigorous manner.

## 3. Results

Table 2 shows the mean and standard deviation of the score, and Cronbach’s alpha for each scale or subscale. The reliability of the scales varied from α = 0.807 to 0.935.

Analysis of variance (ANOVA) was applied to examine if there were any variations on resilience among the three groups (Diagnostic groups: healthy controls, early-stage MCI, late-stage MCI) × 2 (Education level: low, middle to high). Leven’s test was used to assess the equality of variances and indicated no statistically significant differences among the three groups, F (5, 83) = 1.96, *p* > 0.05, η^2^ = 0.071.

Table 3 shows the correlations between resilience, subjective wellbeing, and negative affect in late-stage MCI, early-stage MCI, and healthy individuals.

For Depression and Anxiety, Spearman’s rho correlations were used.

**Figure 1:**
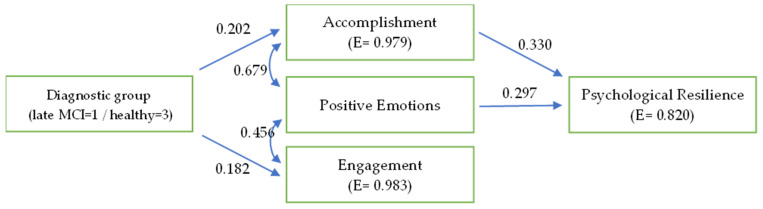
The confirmed path model that shows the differences between late-stage MCI and healthy controls in the profile of relationships between resilience and subjective wellbeing dimensions.

With regard to the first path model confirmed, it has a good fit to the data, χ^2^ (7) = 7.138, *p* > 0.05, CFI = 0.999, SRMR = 0.050, and RMSEA = 0.019 (90% CI: 0.000–0.163). According to the path analysis model (Figure 1), healthy people have higher levels of engagement and accomplishment—the last of which is associated to higher psychological resilience—than people with advanced MCI. Nevertheless, positive emotions are positively related to engagement and accomplishment, and they appear to “lead” to psychological resilience directly and indirectly based on accomplishment, independently of the group type. 

**Figure 2:**
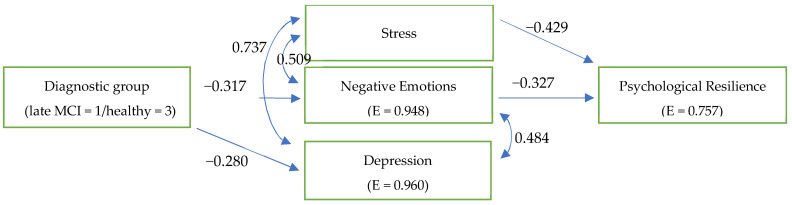
The confirmed path model that shows the differences between late-stage MCI and healthy controls in the profile of relationships between resilience and negative affect dimensions.

On the same diagnostic groups, the confirmed model for the directional relationships between resilience and negative affect also fits well to the data (Figure 2). The S–B χ^2^ (3) = 5.7732, *p* > 0.05, was not significant, CFI = 0.969. However, at this point, it must be noted that RMSEA = 0.130 (90% CI: 0.000–0.286). As the model shows, healthy participants display fewer negative emotions and lower depression symptomatology, as compared to late-stage MCI patients. Moreover, negative emotions are associated with a lower level of resilience. This means that late-stage MCI patients display a lower level of resilience, compared to healthy controls, via this indirect relationship between the group and resilience. Depression is not directly associated with resilience, but only via its positive relationships with both negative emotions and stress. Stress, on the other hand, seems to not be affected by the diagnostic group, but it has a clear negative association with resilience, which seems to be the case for both groups. 

**Figure 3:**
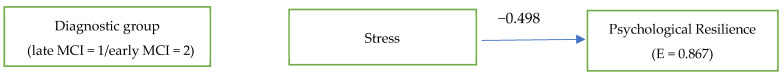
The confirmed path model that shows the similarities between late-stage MCI and early-stage MCI in the profile of relationships between resilience and negative affect dimensions.

The only path model which was confirmed for the two MCI diagnostic groups (Figure 3), S–B χ^2^ (8) = 6.0012, *p* > 0.05, CFI = 1.000, RMSEA = 0.000 (90% CI: 0.000–0.133), underscores that both MCI groups encounter stress that is significantly and negatively associated with their psychological resilience and partially supports the first hypothesis (H_1_).

**Figure 4:**
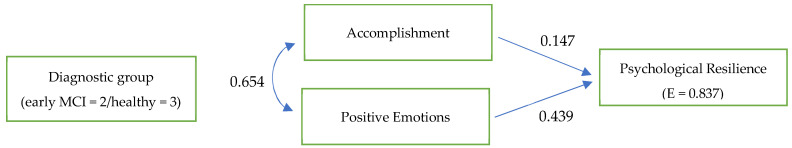
The confirmed path model that shows the differences between early-stage MCI and healthy controls in the profile of relationships between resilience and subjective wellbeing dimensions.

Another path model was confirmed for the healthy controls and the early MCI participants (Figure 4). The indices are good, χ^2^ (9) = 9.183, *p* > 0.05, CFI = 0.999, SRMR = 0.062, RMSEA = 0.018 (90% CI: 0.000–0.146). This path supports the second hypothesis (H_2_) and highlights that both older adults with early MCI as well as healthy individuals experience positive emotions and feelings of accomplishment that can contribute to the enhancement of their resilience levels.

**Figure 5:**
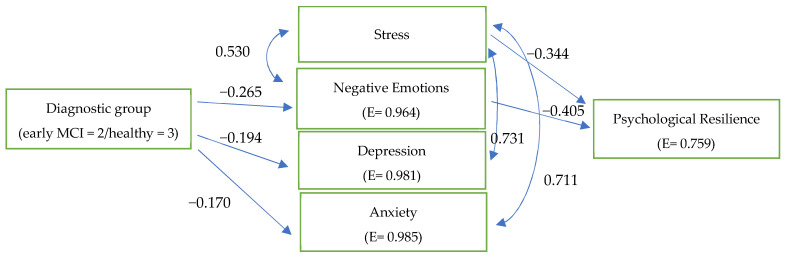
The confirmed path model that shows the differences between early-stage MCI and healthy controls in the profile of relationships between resilience and negative affect dimensions.

Finally, the path model for negative emotions has strong indices as well, S–B χ^2^ (4) = 1.8538, *p* > 0.05, CFI = 1.000, RMSEA = 0.000 (90% CI: 0.000–0.133). It is evident that the early MCI group experiences higher levels of negative emotions, depression, and anxiety compared to the healthy group. However, only negative emotions and stress have negative associations with psychological resilience. Hence, early-stage MCI people seem to experience lower levels of resilience, as compared to healthy controls, mainly via the indirect relationship of group and resilience through negative emotions. Nevertheless, in both groups, stress appears to negatively contribute to resilience, while depression and anxiety seem to relate with resilience only through their highly positive associations with stress (H_2_) (Figure 5).

## 4. Discussion

The purpose of this study was to examine the relationship between the levels of cognitive impairment in older adults with MCI and psychological resilience. More specifically, it is examined that individuals in the advanced stages of mild cognitive impairment (late-stage MCI) may display reduced resilience levels and a distinct pattern of associations between resilience, subjective wellbeing, and negative psychological outcomes, as compared to those in the early-stages of MCI and individuals with normal cognitive function. Additionally, it is examined that those in the initial phases of mild cognitive impairment (early-stage MCI) might show decreased resilience levels and a unique set of relationships between resilience, subjective wellbeing, and negative affect when contrasted with individuals who have no cognitive issues.

The findings of the present study regarding healthy people and late MCI patients show that positive emotions and accomplishment are linked positively to psychological resilience for all of them. Positive emotions play a crucial role for both healthy and late MCI participants, as they enable them to discover a positive meaning in challenging circumstances, learn from them, and promptly recover [85,86]. Notably, there is currently no existing evidence in the literature to support these findings on a late-stage MCI population. On the other hand, the accomplishment levels among late-stage MCI patients are lower compared to those of healthy people. From a previous study, it has been shown that accomplishment, in terms of intention, directedness, and goal-achieving, is associated with less cognitive degradation in aging [87]. The identification and maintenance of life goals has been applied in Alzheimer’s patients to increase their sense of purpose [88], and this was crucial in obtaining higher resilience levels by accepting their current situation and identifying positive features. Furthermore, late-stage MCI patients indicate that their engagement levels are decreased. Sustaining deep involvement in tasks for extended periods is challenging for cognitively impaired individuals [89]. For this reason, interventions on the field of MCI, such as innovative game applications [90] and robot-assistant therapies [91], promote engagement and positive emotional responses, which preserves the effective architecture of the brain’s functional connectivity (FC), enhancing neuro-flexibility (resilience mechanism) in the face of neurodegenerative strain [92].

In terms of negative psychological outcomes, late-stage MCI patients exhibit greater depression and negative emotions than healthy seniors. Severe negative emotions of MCI patients have been linked to poorer psychological resilience. Finally, results show that stress has an inverse relation to resilience without exhibiting any discernible distinction between the two groups. 

There is few research that examines the link of late-stage MCI, negative psychological outcomes, and psychological resilience. These data lack a distinction between MCI stages and the severity of degeneration. An increased risk of MCI is associated with high levels of perceived stress [93], while Smith et al. [68] indicate that negative incidents—as the word refers to adverse experiences—are negatively associated with psychological resilience. The diagnosis of cognitive impairment has an impact on the psychological condition of older adult individuals and allow us to predict their ability to deal with future hardships. Research carried out during the COVID-19 pandemic with Japanese participants who had MCI diagnosis [94] found a link between depressive symptoms and low psychological resilience, although the severity of MCI was not specified. Another study enriches these results by stating that mood disturbances and depressive symptomatology can impact HPA axis dysregulation (main stress mechanism) as well as result in a reduced neuroprotection and neuronal restoration in neurodegenerative diseases [95,96].

Early-stage and late-stage MCI participants exhibit comparable levels of stress, which has an impact on their psychological resilience. This finding supports the previous comparisons between early and late MCI participants, respectively, with healthy people classified into one unified relationship, in which stress has a strong influence on psychological resilience. A recent meta-analysis conducted by Franks and his colleagues [93] reveal a significant association between higher perceived stress and the risk of MCI. At a biological level, it is not clear whether oxidative stress precedes or is a result of the degeneration process, although it has been found that it is involved in the cellular injury process that concur in neurodegenerative diseases [97,98] and, particularly, in MCI [99]. Stress is also associated with lower levels of resilience, but only when maladaptive coping strategies are implemented [100]. Further experimental studies should be conducted for MCI patients to reveal a clearer relationship between the cognitive state, stress levels, and psychological resilience.

The findings regarding early MCI patients and people with no cognitive impairment showed that both groups present the same levels of accomplishment and positive emotions, which are positively related to their psychological resilience. In alignment with the broaden-and-build theory [85], positive emotions allow a person to broaden his/her mindset and attribute beneficial meaning to past experiences, “building” resources for psychological resilience in this way. Taherkhani and her colleagues [101] also demonstrate that positive thinking can result in higher psychological resilience. Early MCI patients realize the existence of certain modest cognitive impairments, but they are nevertheless able to discover counterbalancing strategies and regulate their emotions to address their issues in the same manner that healthy individuals do. However, there is currently insufficient scientific evidence to establish well-defined associations between positive emotions, accomplishment, and psychological resilience specifically among early MCI patients. 

Finally, a comparable picture is shown by the relationship of early MCI people with the healthy ones regarding the negative psychological outcomes. Early MCI participants, report higher levels of depression and negative emotions as well as of anxiety. In previous research, patients with MCI are more likely to develop a depressive or anxiety disorder [102,103], while anxiety has been presented as an early indicator of Alzheimer’s disease and associated dementias [104,105]. Moreover, it is proven that psychological disorders, such as depression, render the brain more susceptible to neuropathological changes of Alzheimer’s disease [106]. As previously mentioned, anxiety, depression, and negative emotions are common feelings in the beginning of neurodegenerations, and impacts resilience levels by influencing the stress mechanisms in the brain [95,96]. 

To sum up, the investigation unveiled both commonalities and disparities among the observed variables within the three distinct cohorts. Specifically, individuals categorized as possessing good health and those being in the initial stages of mild cognitive impairment (early-stage MCI) exhibited comparable levels of achievements, positive emotions, and stress. Additionally, positive emotions and stress levels were found to be similar between the healthy individuals and those in the advanced stages of MCI (late-stage MCI). Furthermore, no significant differences in stress levels emerged when comparing the two MCI subgroups (early and late stages). On the contrary, discernible differences were identified in negative emotions, depression, and anxiety levels between the healthy subjects and those with early-stage MCI. Also, disparities were evident in achievement, engagement, negative emotions, and depression levels when comparing healthy participants to those with late-stage MCI.

Taking into account the study’s correlational nature, it is valuable to delve deeper into understanding the specific direction of associations depicted within the path models. The research carefully investigated how certain subjective wellbeing factors and experiences of distress impacted the psychological resilience level of MCI patients. However, the reverse associations of the aforementioned variables obtained in the path models could be further studied. For example, Ovaska–Stafford and colleagues [107] in an extended literature review ascertained a strong association between higher levels of resilience and fewer depressive symptoms, anxiety, and negative emotions. Additionally, they deduced that resilience could account for a significant 22.5% variation in Quality of Life (QOL) experienced by individuals dealing with neurodegenerative diseases. These relationships warrant further investigation to precisely determine through intervention studies the directions and the patterns of these relations. 

These findings expand the theoretical framework of psychological resilience, including positive emotions, feelings of accomplishment, engagement capacities, negative emotions, depression, anxiety, and stress in MCI people. 

## 5. Implications

Some potential implications can be formulated based on the findings of this investigation. To begin with, there is strong evidence that positive emotions and actions serve as an effective defense against the progression of neurodegeneration. At the same time, negative emotions, anxiety, depressive feelings, and thoughts deteriorate the neurocognitive backdrop and accelerate neurodegeneration. At this point, it should be noted that the current study’s late MCI patients had a lower educational level in contrast to early and healthy participants, which has been demonstrated as a risk factor for developing fast and severe types of cognitive impairment [108,109]. 

In line with these findings, psychological interventions that aim to increase positive emotions and accomplishments should be an integrant in day-to-day settings as well as in daily health centers. The main purpose of such interventions in MCI patients would be to build positive resources, improve their psychological resilience, and help them cope with the psychological effects of living with a neurodegenerative disease, providing them with positive meaning in their lives. Participants will be encouraged to work on daily activities and increase their cognitive flexibility by discovering new strategies to solve everyday challenges and feel competent. 

Individuals with both early and late MCI diagnoses can attend group sessions to receive therapy for their stress, depression, negative emotions, and thoughts. This is a critical topic since psychological challenges might limit the potential positive effects of the interventions and are able to expedite the progression of the degeneration. Furthermore, it is significant that these mood difficulties, such as anxiety and depression, can have a neurobiological impact on MCI patients, thereby complicating the structure of interventions for this population [110]. Particularly, in early MCI patients, an intervention for anxiety could be highly advantageous, as this group tends to exhibit high levels of anxiety. The anticipated results of the intervention might involve inquiring stress management practices and relaxation techniques as well as involvement in physical and social activities. 

Overall, the above evidence can be incorporated partially or as a whole in interventions for the reinforcement of psychological resilience of older MCI adults. Firstly, it is suggested to incorporate psychoeducation to provide important information about psychological resilience and thoroughly explain the intervention purposes to the participants. Subsequently, older adults are advised to face their psychological difficulties (depression, anxiety, stress, negative emotions) by reframing their negative beliefs about their cognitive abilities and learn coping strategies for regulating their emotions and behaviors. The intervention program would adopt a comprehensive approach, including social support networks, physical exercise groups, and recommendations of lifestyle habits, mindfulness-based techniques, and cognitive-behavioral therapy (CBT) exercises. All the above would aim to increase the positive emotions and feelings of accomplishment of people at risk. Finally, to enhance the intervention outcomes, it is suggested that family members and caregivers actively participate in the intervention by following the training and education for MCI caregivers and create a supportive environment for the MCI individuals. 

## 6. Limitations

There are certain limitations in this study that should be noted for future research. First, more emphasis should be given to seniors’ comprehension of the concepts employed in the study. The survey was a self-reported measure, and participants were encouraged to ask the researchers about confusing items. This factor, along with the length of the questionnaires, may have contributed to their mental fatigue. Moreover, in this study, a female predominance was observed, and late-stage MCI patients with low educational levels numerically exceeded those with higher educational levels. As a result, future research should be focused on balanced gender samples, with diverse educational backgrounds and varying stages of MCI.

In terms of the study’s sample size, the three groups were rather small, preventing the detection of stronger associations between the examined variables. Moreover, only self-reported measures were used, and the findings were not cross-referenced with additional neuroimaging techniques and biomarkers. Furthermore, it should be noted that this study presents some cross-sectional data, which highlight a direction but not causality. To establish definite patterns, further research with a larger representation of the three groups should be conducted as well as the use of different objective indicators. Additionally, the subtypes of MCI diagnosis can be used in future research to further describe the sample characteristics. Longitudinal and experimental studies on this topic would yield a more comprehensive understanding of the interplay between these constructs and allow the structure of more efficient counseling programs to help people at risk. 

## 7. Conclusions

This study highlights some significant relations between mild cognitive impairment, subjective wellbeing factors, depression, anxiety, stress, and psychological resilience. Results suggest that early MCI participants experience comparable levels of positive emotions and feelings of accomplishment as their healthy counterparts. This implies that positive emotions and sense of accomplishment remain relatively preserved. However, as MCI proceeds in late stage, there is a substantial drop in feelings of accomplishment, which has a detrimental influence on psychological resilience.

Late-stage MCI patients are vulnerable to negative emotions and stress, diminishing their resiliency. On the other hand, early-stage MCI participants experience greater negative psychological burden, which undermines their psychological resilience. 

## Figures and Tables

**Table 1:** Mean and S.D. of the groups.

Demographic Variables	Late MCI (n = 28)	Early MCI (n = 31)	Healthy (n = 30)
Male (n)	9	5	9
Female (n)	19	26	21
Age (years) M (S.D.)	75.68 (5.07)	73.39 (7.65)	69.60 (5.30)
Education			
Low (%)	19 (67.9%)	6 (19.4%)	3 (10%)
High (%)	9 (32.1%)	25 (80.6%)	27 (90%)

**Table 2:** Descriptions for the three groups of the sample and reliability values of all scales.

Assessment Tools	Late MCI	Early MCI	Healthy	Cronbach’s Alpha
Brief Resilience Scale	3.07 (±0.99)	3.18 (±0.67)	3.45 (±0.68)	0.807
PERMA Profiler	6.75 (±0.87)	6.68 (±0.92)	6.63 (±0.95)	0.847
Positive Emotions	7.15 (±1.39)	6.80 (±1.71)	7.06 (±1.55)	0.844
Engagement	6.48 (±1.64)	6.76 (±1.67)	7.11 (±1.70)	0.565
Accomplishment	6.40 (±1.62)	6.80 (±1.57)	7.22 (±1.66)	0.759
Negative Emotions	5.50 (±2.89)	5.17 (±1.67)	4.19 (±1.63)	0.559
Dass-21	0.61 (±0.55)	0.66 (±0.59)	0.47 (±0.45)	0.935
Depression	0.73 (±0.67)	0.71 (±0.73)	0.43 (±0.49)	0.865
Anxiety	0.40 (±0.54)	0.46 (±0.63)	0.25 (±0.40)	0.866
Stress	0.69 (±0.62)	0.80 (±0.55)	0.74 (±0.58)	0.855

**Table 3:** Correlations between resilience and the other variables of interest in late-stage mild cognitive impaired (MCI) patients’ group, early-stage MCI patients’ group, and healthy individuals.

		RES	POSEM	ENG	REL	MEAN	ACC	NEG	LON	PHY	HAP	DEP	ANX	STRESS
RES	Late MCI	1	0.495 **	0.450 *	Non sig.	0.445 *	0.510 **	−0.394 *	−0.431 *	Non sig.	0.580 **	−0.540 **	−0.524 **	−0.526 **
	Early MCI	1	0.456 *	Non sig.	Non sig.	0.420 *	Non sig.	−0.453 *	−0.381 *	0.366 *	Non sig.	−0.376 *	−0.147	−0.421 *
	Healthy	1	0.621 **	Non sig.	0.554 **	0.387 *	0.507 **	-0.638 **	−0.598 **	0.421 *	0.607 **	−0.721 **	−0.508 **	−0.672 **

* *p* < 0.05, ** *p* < 0.01. RES: Resilience, POSEM: Positive Emotions, ENG: Engagement, REL: Positive Relationships, MEAN: Meaning, ACC: Accomplishment, NEG: Negative Emotions, LON: Loneliness, PHY: Physical Health, HAP: Happiness, DEP: Depression, ANX: Anxiety, STRESS: Stress.

## Data Availability

Data available upon duly justified request.
